# The Impact of Adjunctive Aripiprazole on Olanzapine‐Induced Metabolic Adverse Effects in Patients With Schizophrenia: A Systematic Review

**DOI:** 10.1002/npr2.70046

**Published:** 2025-08-31

**Authors:** Stephen Simmons, Soban Sadiq

**Affiliations:** ^1^ Kent and Medway Medical School University of Kent Canterbury UK

**Keywords:** adjunctive, aripiprazole, metabolic adverse effects, olanzapine, schizophrenia

## Abstract

**Background:**

Olanzapine is a second‐generation atypical antipsychotic drug which is commonly used in the treatment of schizophrenia. It has been associated with metabolic adverse effects such as weight gain, hyperglycaemia, dyslipidaemia, and this has been shown to contribute to the reduction of life expectancy of patients with schizophrenia. This systematic review aimed to assess whether adjunctive aripiprazole is effective at reducing metabolic adverse effects caused by olanzapine.

**Methods:**

A systematic review was conducted for this study. A systematic search strategy was developed, recorded, and applied to multiple databases. The literature search found a total of 853 results with the final inclusion of 7 research articles. Based on specific inclusion and exclusion criteria, a wide range of study designs were included in the review, such as randomized control trials (RCTs), open label trials, and case series. Key outcomes were identified, which included glucose levels, lipid profile, body weight, BMI, and waist circumference. The results were recorded and analyzed using narrative synthesis.

**Results:**

Statistically significant decreases in fasting triglycerides were consistent across multiple studies. Adjunctive aripiprazole shows potential weight loss benefits, with some studies reporting significant reductions in weight and BMI. Effects on cholesterol and fasting glucose showed reductions, and others showed minimal or no impact. Psychiatric symptom control remained stable in most studies, suggesting that aripiprazole does not negatively affect schizophrenia symptoms while potentially providing metabolic advantages.

**Conclusion:**

Adjunctive aripiprazole had variable effects on metabolic parameters in patients on olanzapine therapy; however, reductions in triglycerides appeared consistent among most of the data, and some studies reported significant weight loss. This highlighted that aripiprazole does have some effect in reducing metabolic adverse effects caused by olanzapine.

## Abstract 2

PRISMA flow diagram illustrating the selection process for studies included in the systematic review. This systematic review evaluates the efficacy of adjunctive aripiprazole in reducing metabolic adverse effects in patients with schizophrenia treated with olanzapine. The findings suggest that adjunctive aripiprazole may provide modest improvements in weight and triglyceride levels, though evidence on other metabolic parameters remains inconclusive.

## Graphical abstract

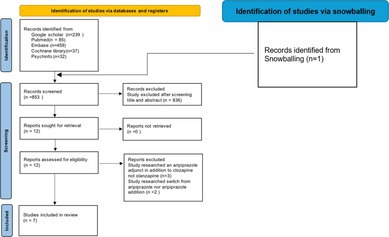

## 1 Introduction

Schizophrenia is a primary psychotic disorder which is characterized by numerous chronic symptoms such as delusions, hallucinations, and disorganized thoughts and behavior [1]. The pathophysiology of schizophrenia is understood to be caused by an increase of dopamine and dopamine receptors in the mesolimbic dopamine pathway; this causes the positive symptoms seen, such as delusions and hallucinations [2]. The disease has an early onset, and due to its relapse‐remitting nature, it can have a major disabling impact for patients and their families. Pharmacological treatment of schizophrenia includes prescribing atypical and typical antipsychotics. Atypical antipsychotics are a group of medications that act as dopamine antagonists postsynaptically and serotonin agonists presynaptically, overall working to reduce dopaminergic activity [3]. Atypical antipsychotics are preferred to typical antipsychotics due to the lower risks of extra‐pyramidal side effects and increased efficacy [4]. An example of a commonly used atypical antipsychotic is olanzapine. Olanzapine is an effective treatment of schizophrenia; however, it can cause metabolic adverse effects to patients. The metabolic adverse effects of olanzapine include weight gain, increased waist circumference, dyslipidaemia, type 2 diabetes mellitus, and increased body mass index (BMI) contributing to metabolic syndrome [5]. A study found that 67% of participants experienced weight gain of more than 7% after 12 months of olanzapine treatment [6]. In the same study, the mean total cholesterol values increased by 18.7%, and the mean BMI values increased from 24.4 to 28.1 after 12 months of treatment with olanzapine. Avoiding or mitigating the metabolic adverse effects, such as increased total cholesterol, is very important as increased total cholesterol levels were significantly associated with elevated risk of cerebrovascular disease and ischaemic heart disease [7].

Aripiprazole is another atypical antipsychotic. Studies have shown that the use of an aripiprazole adjunct reduces the metabolic adverse effects of olanzapine [8, 9]. However, due to the heterogeneity of the data, there is not a definitive picture of the effect of adjunctive aripiprazole. Therefore, the purpose of this review is to assess whether an aripiprazole adjunct reduces the olanzapine‐induced metabolic adverse effects in patients with schizophrenia.

### 1.1 Background and Context

The metabolic adverse effects of antipsychotic drugs like olanzapine are very impactful on patients. It is well documented that there is an increased prevalence of cardiovascular risk factors in patients living with schizophrenia [10]. This is in part due to these metabolic adverse effects, such as weight gain and raised triglyceride and total cholesterol levels, of antipsychotic medication [11]. As a result, people living with schizophrenia are twice as likely to die from CVD compared to those in the general population [12]. It is also documented that the life expectancy of people living with schizophrenia is reduced by over 20 years [13] and 60% of this excess mortality was caused by physical illness [14]. Deaths from cardiovascular disease are the major contributor to this excess mortality caused by physical illness and it has been suggested that the gap in mortality between people with schizophrenia and the general population may be widening even further [15]. This highlights that to reduce these metabolic adverse effects, pharmacological management such as switching antipsychotics may be an option but may not always be possible, as the clinician must manage the risk of relapse versus the adverse metabolic effects. Therefore, it is important for clinicians to have other options to mitigate these risks. Adjunctive pharmacological options may be effective in mitigating or treating these adverse effects [16]. Currently, there is no recommendation from the National Institute of Clinical Excellence (NICE) on adjunctive pharmacological therapy. The data from 3 RCTs state that an aripiprazole adjunct would appear to be a safe and potentially effective option to reduce weight gain in patients prescribed olanzapine without causing detrimental effects on psychiatric symptoms. These 3 RCTs investigated the effects of adjunctive aripiprazole added to patients taking olanzapine or clozapine. Of these 3 RCTs, only 1 focused on olanzapine‐treated patients. Based on the single randomized control trial (RCT), it can be stated that an aripiprazole adjunct has some effect on olanzapine‐associated metabolic adverse effects. In regard to weight and BMI reduction, following 4 weeks of treatment with aripiprazole, there was a significant decrease in weight compared to placebo treatment and a parallel decrease in BMI [8]. Total serum cholesterol, high‐density lipoprotein (HDL) cholesterol, low‐density lipoprotein (LDL) cholesterol, and fasting glucose did not change significantly, but there were significant decreases in triglyceride levels [8]. Another RCT does show similar findings in lipid levels, with the study demonstrating a statistically significant decrease in serum fasting triglycerides [9]. However, the same study also shows significant weight gain due to the addition of aripiprazole and an increase in waist circumference; however, the results were statistically insignificant [9].

Due to the varied evidence, this systematic review has searched databases to find a broad range of studies to assess the metabolic advantages of adjunctive aripiprazole on olanzapine‐treated patients.

### 1.2 Research Question

The research question “Does the use of aripiprazole as an adjunct with olanzapine reduce the metabolic adverse effects in patients with schizophrenia?” was formulated using the PICO tool as seen in Table 1. The PICO tool was used and provided a structured approach to derive a well‐defined research question [17].

**TABLE 1:** Development of the research question.

PICO PICO information: http://www.ncbi.nlm.nih.gov/pmc/articles/PMC3430448/	
Population?	Adults diagnosed with schizophrenia treated with Olanzapine
Intervention?	Aripiprazole
Comparison?	None
Outcomes?	Reduction of Olanzapine induced metabolic adverse effects
My research question	Does the use of Aripiprazole as an adjunct with Olanzapine reduce the metabolic adverse effect in schizophrenic adult patients?

### 1.3 Aim

The aim of this research was to complete a systematic review on the relevant literature to assess if the use of aripiprazole as an adjunct with olanzapine reduces the metabolic adverse effects in patients with schizophrenia.

### 1.4 Objectives

- To identify the effectiveness of adjunctive aripiprazole through a comprehensive synthesis of existing literature.
- To assess clinical trials to highlight whether aripiprazole is effective at reducing olanzapine‐induced metabolic adverse effects, by evaluating metabolic parameters such as weight, BMI, waist circumference, lipid levels, and glucose levels.
- To assess gaps in current published research and recommend areas for future investigation that may aid in understanding the impact adjunctive aripiprazole has on olanzapine‐associated metabolic adverse effects.

### 1.5 Search Strategy

A key aspect of a systematic review is identifying pertinent studies in an unbiased and comprehensive way. Clearly reporting the search strategy allows for an assessment of its quality and reliability [18]. The Preferred Reporting Items for Systematic Reviews and Meta‐analyses (PRISMA) tool for systematic reviews methods section was used to develop a search strategy for this study where this section discussed items 5 to 8 [19] (Supporting Information).

#### 1.5.1 Eligibility Criteria

The inclusion and exclusion criteria used for the search strategy were developed in conjunction with the research question formed using the PICO framework as seen in Table 1 [17]. Studies were included if they were published in the last 20 years, published in the English language, included adult schizophrenia patients, and were primary quantitative studies. Studies were excluded if they were non‐adult studies or if they were qualitative. It was concluded that to be eligible, studies must include a population of patients with schizophrenia as these patients are likely to be taking antipsychotic drugs like olanzapine [20].

#### 1.5.2 Information Sources

The database selection was based on which databases would be most relevant to the research question. The databases selected were Embase (Ovid), Google Scholar, Psych Info, PubMed, and Cochrane Library.

#### 1.5.3 Search Strategy Development

The research question developed is as follows: does the use of aripiprazole as an adjunct with olanzapine reduce the metabolic adverse effect in patients with schizophrenia? This was created using the PICO framework as seen in Table 1 [17]. Keywords such as olanzapine, aripiprazole adjunct, metabolic adverse effects, and schizophrenia were identified and used in the search. Synonyms were also used to prevent papers from being excluded due to differences in the drug brand name and generic name, such as zyprexa and olanzapine. A search string was formulated and can be seen in the Supporting Information. Boolean operators were used to search the databases, such as OR and AND. The advanced search option was used for each of the five search engines, and the search string was applied. Ovid, PubMed, Cochrane Library, PsychInfo, and Google Scholar were the databases used. The snowballing technique was used to identify one additional study.

#### 1.5.4 Selection Process

Studies were assessed to determine if they met the inclusion criteria. This involved an initial review of many aspects of the article such as the title and abstract, study design, the age of the study, patient demographics, interventions, and the outcomes measured. A review of the discussion, interpretation of results, and limitations of the study was also carried out.

**FIGURE 1:**
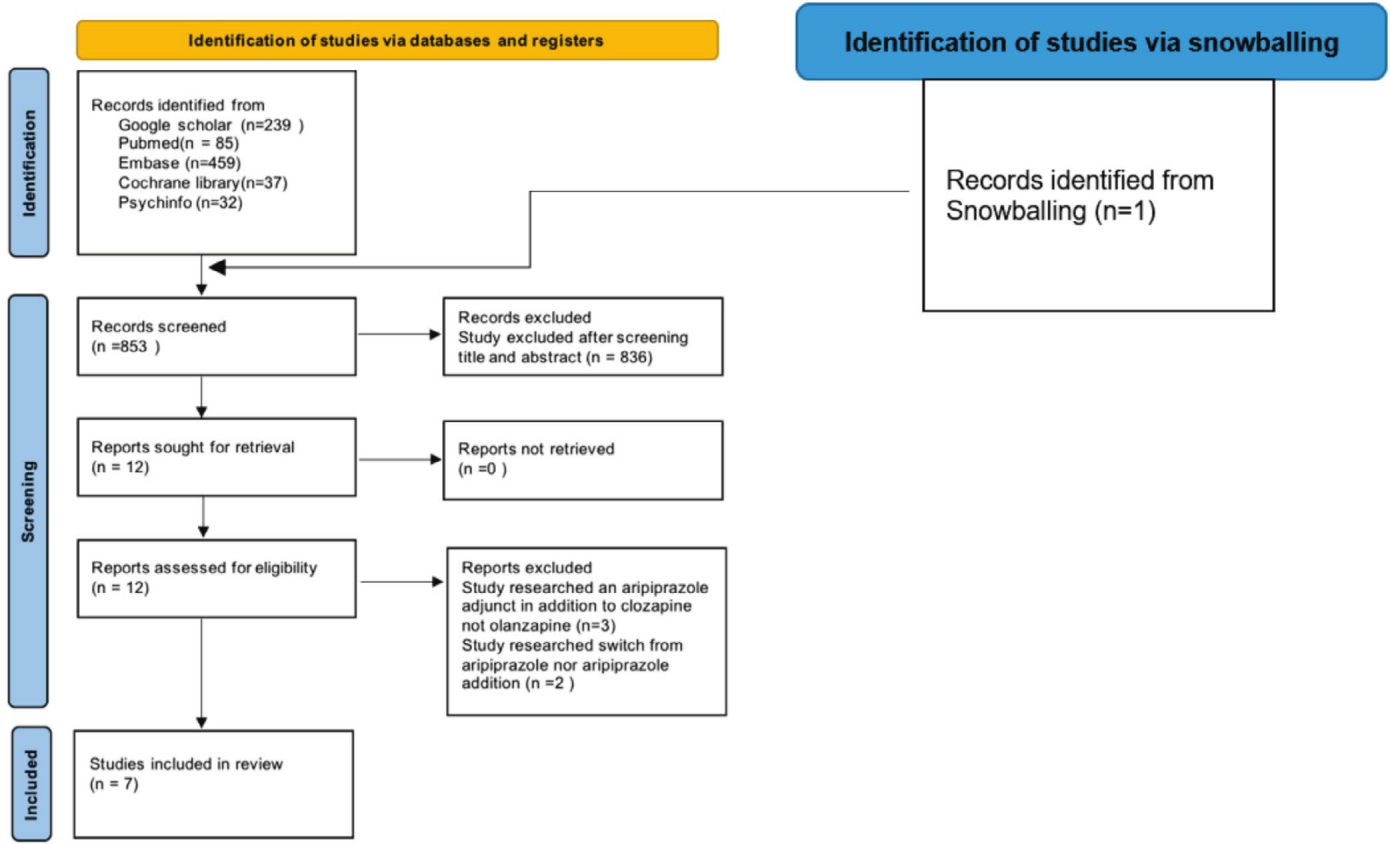
PRISMA flow diagram illustrating the selection process for studies included in the systematic review. Seven studies met the inclusion criteria and were included in the final review.

The PRISMA flow diagram (Figure 1) is a method of recording different stages of the systematic literature search. This enhances the reliability and quality of systematic reviews. The four stages of the flow diagram are identification, screening, eligibility, and inclusion [19]. Seven studies met the inclusion criteria and were included in the final review (Table 2).

**TABLE 2:** Table of final studies included in this systematic review.

Study 1. D. C. Henderson, X. Fan, P. M. Copeland, et al., “Aripiprazole Added to Overweight and Obese Olanzapine‐Treated Schizophrenia Patients,” *Journal of Clinical Psychopharmacology* 29, no. 2 (2009): 165–169.
Study 2. B. Gupta, K. S. Chee, L. Q. Neo, et al., “Effect of Aripiprazole as an Adjunct to Atypical Antipsychotics on Weight and Metabolic Profile: A 12‐Week Open‐Label Trial,” *Therapeutic Advances in Psychopharmacology* 11 (2021): 1–14.
Study 3. L. J. Wang, S. C. Ree, Y. S. Huang, C. C. Hsiao, and C. K. Chen, “Adjunctive Effects of Aripiprazole on Metabolic Profiles: Comparison of Patients Treated With Olanzapine to Patients Treated With Other Atypical Antipsychotic Drugs,” *Progress in Neuro‐Psychopharmacology & Biological Psychiatry* 40 (2013): 260–266.
Study 4. G. Sulejmanpasic, S. Bise, F. Pepic, and A. Toskic, “P.105 Adjunctive Treatment of Aripiprazole to Olanzapine for Weight Reduction in Patients with Schizophrenia,” *European Neuropsychopharmacology* 29 (2019): S90.
Study 5. T. Jia, X. Len, Z. Pi, Z. Hong, J. Feng, and C. Ma, “Effect of Aripiprazole Combined With Olanzapine on the Clinical Efficacy of Schizophrenia,” *Farmácia* 70, no. 3 (2022): 550–556.
Study 6. S. Englisch, A. Weinbrenner, D. Inta, and M. Zink, “Aripiprazole for the Management of Olanzapine‐Induced Weight Gain,” *Pharmacopsychiatry* 42, no. 4 (2009): 166–167.
Study 7. S. M. Khaleel, M. M. Khalaf, and M. S. Hasan, “Potential Mitigation of Olanzapine‐induced Derangement of Blood Sugars by Adding Aripiprazole to the Therapeutic Regimen of patients with Schizophrenia: A Follow‐up Study,” *Systematic Reviews in Pharmacy* 11, no. 8 (2020): 183–188.

## 2 Methods

This systematic review was registered in PROSPERO (Registration ID: CRD420251057301).

A systematic review of research on how aripiprazole as an adjunctive therapy affects the metabolic adverse effects of olanzapine was undertaken. The PRISMA checklist, which can be seen in the Supporting Information, has been used to ensure a robust method has been carried out.

### 2.1 Data Collection Process

Data was collected manually by one author and reviewed by another author and inserted into two tables (Tables 3 and 4). The data items sought were based on the research question, review of the existing literature, and the inclusion and exclusion criteria. Data extracted included the study design, study length, sample size, participant characteristics, inclusion and exclusion criteria, the dose of both olanzapine and aripiprazole, and the statistical analysis used.

**TABLE 3:** Individual study results: Studies denoted as 1–7.

Metric	1	2	3
Weight/BMI	Weight	Weight	Weight
	−2.9 lbs (1.3 kg)	+0.43 kg	−2.2 kg
	*p* = 0.003	*p* = 0.325	*p* = 0.026
	BMI	BMI	BMI
	−0.4 kg/m^2^	−0.007 kg/m^2^	−0.8 kg/m^2^
	*p* = 0.003	*p* = 0.681	*p* = 0.028
Waist circumference	−0.76 cm	+0.73 cm	Not recorded
	*p* = 0.063	*p* = 0.510	
Fasting triglycerides	−51.7 mg/dL	−0.34 mmol/L	−74.0 mg/dL
	*p* = 0.001	*p* = 0.001	*p* = 0.006
Fasting cholesterol (total, HDL and LDL)	Total	Total	No results for Olanzapine group in isolation
	‐3 mg/dL	−0.18 mmol/L	
	*p* = 0.208	*p* = 0.138	
	HDL	HDL	
	0.4 mg/dL	+0.05 mmol/L	
	*p* = 0.999	*p* = 0.079	
	LDL	LDL	
	−0.2 mg/dL	−0.07 mmol/L	
	*p* = 0.665	*p* = 0.469	
Fasting glucose	−2 mg/dL	+0.15 mmol/L	No results for Olanzapine group in isolation
	*p* = 0.704	*p* = 0.17	
Psychiatric control score using PANSS	No significant difference between groups	+1	No results for Olanzapine group in isolation
		*p* = 0.611	

Metric	4	5	6	7
Weight/BMI	No data Claims significant change in both	Not recorded	Weight −0.7 kg *p* < 0.05 BMI −0.2 kg/m^2^ *p* < 0.05	Weight reduction not stated BMI −0.73 kg/m^2^ *p* = 0.000
Waist circumference	Not recorded	Not recorded	Not recorded	Not recorded
Fasting triglycerides	No data Claims significant decrease	Slight decrease data in bar graph so cannot provide exact figures *p* > 0.05	No data Claims no significant change	Not recorded
Fasting cholesterol (total, HDL and LDL)	No data Claims significant change HDL and LDL not recorded	Slight decrease data in bar graph so cannot provide exact figures *p* > 0.05 HDL and LDL not recorded	No data Claims no significant change	Not recorded
Fasting glucose	No data Claims significant change	Slight decrease Data in bar graph so cannot provide exact figures *p* > 0.05	No data Claims no significant change	−6.7 mg/dL *p* = 0.000
Psychiatric control score using PANSS	No data Claims no significant change	Significant change after starting treatment *p* < 0.05 note patients are presenting with schizophrenia for the first time	No specific data States non significant improvement in PANSS score	Not recorded

**TABLE 4:** Characteristics of the selected studies.

	1	2	3
Type of study	Randomized controlled trial	Open labeled trial	Open labeled trial
Objective of study	To observe the effect of aripiprazole when added to obese olanzapine treated schizophrenic patients	To evaluate the effect of adjunct use of aripiprazole at a fixed dose of 5 mg on metabolic profile and weight in patients stabilized on atypical antipsychotics olanzapine, clozapine and risperidone	This study aims to determine whether adjunctive treatment with aripiprazole improves the metabolic parameters and clinical symptoms among patients under a pre‐existing treatment with atypical antipsychotics, including olanzapine
Number of participants	15. 1 patient dropped out one for medication changes making them ineligible	18 patients on olanzapine, 55 overall	43. 3 dropped out of the original 46
Inclusion criteria	Outpatients between the ages of 18 and 65 with a diagnosis of schizophrenia or schizoaffective disorder were included after providing written consent. Subjects were eligible for the study if their body mass index (BMI) was ≥ 30 kg/m^2^; or ≥ 27 kg/m^2^ with other risk factors (treatment for hypertension of blood pressure (BP) > 140/90 mmHg; lipid abnormalities: total cholesterol ≥ 200 mg/dL, triglyceride ≥ 150 mg/dL; or fasting glucose ≥ 100 mg/dL). Subjects had to be maintained on a stable dose of olanzapine for at least one month	The study recruited outpatients aged 21–65 years with a diagnosis of schizophrenia or schizoaffective disorder. The subjects were required to be on stable doses of atypical antipsychotics, olanzapine, clozapine, or risperidone, for at least 1 month. Antipsychotic polypharmacy was permitted, as long as there was only one of the above three atypical antipsychotics. At baseline, subjects were required to have a BMI ≥ 25 kg/m^2^ (i.e., overweight and above) or ≥ 7% increase in weight from pre‐antipsychotic treatment	Eligible patients at an out‐patient department were selected for this study if they: (1) aged between 18 and 65 years, (2) were assessed using the Mini International Neuropsychiatric Interview and diagnosed with a psychotic disorder (schizophrenia, schizoaffective disease disorder, or bipolar disorder) as defined by the DSM‐IV (3) had been treated with an atypical antipsychotic drug other than aripiprazole, and were maintained on a stable dose of the same antipsychotic for at least one month prior to study start, and (4) met any criteria of metabolic abnormality (including being overweight, obesity, hyperglycaemia, or dyslipidaemia) as judged by investigators to be associated with antipsychotic treatment
Exclusion criteria	Patients with active substance abuse, pregnancy, significant medical illness (unstable cardiac disease, malignancy, severe liver or renal impairment), and unstable psychiatric illness (CGI's severity of illness question of 5 or greater) were excluded from the study	The study excluded subjects who had a previous allergy to aripiprazole or contraindication to the use of aripiprazole. Participants with current substance misuse or those non‐adherent to current prescribed medications were excluded. It was required that subjects had no major or unstable medical or neurological illness (such as uncontrolled diabetes and hypertension) and were not using any medications for weight loss during the preceding month. Participants with serious suicidal thoughts or who posed a serious risk of harm to self or to others were excluded. The study did not recruit women who were pregnant or breastfeeding. Participants who had clinically significant abnormalities on enrolment examination and screening that required active intervention, that is, initiation of lipid lowering agent or antidiabetic medication, were also excluded	This study excluded patients if they were (1) women who are pregnant, breast feeding, or planning to conceive, (2) a psychotropic regimen (other than that for aripiprazole) that needs to be adjusted during the study, (3) patients with a significant medical illness, acute psychosis, acute suicide ideation, or any acute psychiatric condition that may require emergency intervention
Length of study	10 weeks, 4 week treatment arms with a 2 week adjunctive treatment washout	12 weeks	8 weeks
Metabolic outcome measured included in this systematic review	Weight BMI Waist circumference Fasting triglycerides Fasting cholesterol (total HDL and LDL) Fasting glucose PANSS score	Weight BMI Waist circumference Fasting triglycerides Fasting cholesterol (total HDL and LDL) Fasting glucose PANSS score	Weight BMI Fasting triglycerides Fasting cholesterol (total HDL and LDL) Fasting glucose PANSS score
Dose of olanzapine + aripiprazole assessed	Olanzapine 22 mg Aripiprazole 15 mg	Olanzapine 11.9 mg Aripiprazole 5 mg	Olanzapine 10 mg Aripiprazole 10.4
Patient demographic	Age—The mean age of subjects was 49 ± 8 years old Sex—ten (67%) were male and 5 were female (33%) Ethnicity Three subjects were African American (20%) and twelve (80%) were Caucasian	Age—mean age: 37.7 Sex—7 female 11 male Ethnicity—13 Chinese 3 Malay 1 Indian 1 other	Age—mean age: 37.8 ± 10.8 Sex—16 males and 27 females Ethnicity—all patients were Taiwanese
Statistical analysis performed	SPSS	SPSS	SPSS

	4	5	6	7
Type of study	Randomized control trial	Randomized control trial	Case series	Case series
Objective of study	The aim was to compare the effectiveness and tolerability of the adjunctive treatment of aripiprazole to olanzapine with the olanzapine monotherapy for weight reduction in patients with schizophrenia	This study aims to observe the effect of olanzapine combined with aripiprazole in the treatment of schizophrenia and to explore the improvement of adverse reactions caused by the two drugs	This study aims to observe is adjunctive aripiprazole affects olanzapine induced metabolic adverse effects	This study aims to investigate whether the addition of aripiprazole to olanzapine therapy will improve glycemic control and body weight in olanzapine treated patients with schizophrenia and if there are any gender differences between patients regarding susceptibility to metabolic disturbances due to antipsychotics
Number of participants	8 patients 4 receiving olanzapine 4 receiving placebo	68	4	29
Inclusion criteria	None listed	Participants of this study were included if (1) they had a diagnosis in line with the diagnostic criteria for schizophrenia in the Guidelines for Clinical Diagnosis and Treatment—Psychiatry Sub Volume of Chinese Medical Association; (2) first onset, course of disease ≤ 36 months; (3) no previous history of antipsychotic drug use; (4) Positive and Negative Syndrome Scale (PANSS) score ≥ 60 points; (5) no contraindications for the research drugs; (6) with the informed consent of patients and their families	Not explicitly stated but all patients were diagnosed with schizophrenia as per the DSM IV criteria and all patients had experienced an increase in BMI with the mean increase being 2.8	Participants of this study were schizophrenic patients who were stable on olanzapine monotherapy on a daily dose of 10 mg for at least three months prior to the enrollment
Exclusion criteria	None listed	Criteria: (1) severe heart, liver, kidney and other organic diseases; (2) generalized developmental disorders and mental retardation; (3) history of alcohol and drug dependence; (4) pregnant and lactating women; (5) hematological, neurological and infectious diseases; (6) combined with malignant tumor; (7) thyroid disease, diabetes and other endocrine and metabolicN diseases; (8) application of drugs affecting thyroid hormone level in recent 3 months; (9) giving up treatment halfway	Not explicitly stated	patients with dyslipidemia, diabetes or other endocrine disorder, patients with schizophrenia managed with polypharmacy, patients with renal and/or hepatic ailments, and pregnant or lactating mother. Schizophrenic patients managed with drugs known to affects glucose tolerance such as (β‐adrenoceptor blockers, thiazides diuretics etc.) were also excluded
Length of study	8 weeks	8 weeks	8 weeks	8 weeks
Metabolic outcome measured included in this systematic review	BMI Weight Fasting triglycerides Fasting cholesterol (total HDL and LDL) Fasting glucose PANSS score	Fasting triglycerides Fasting cholesterol (total) Fasting glucose PANSS score	Weight BMI Fasting triglycerides Fasting cholesterol (total HDL and LDL) Fasting glucose PANSS score	Weight BMI Fasting glucose
Dose of olanzapine + aripiprazole assessed	Olanzapine 20 mg Aripiprazole 15 mg	Olanzapine not explicitly stated but all does were between10 and 20 mg Aripiprazole 10 mg	Olanzapine 11.3 mg Aripiprazole 13.8 mg	Olanzapine‐ not explicitly stated but maintained through out Aripiprazole 10 mg
Patient demographic	Not listed	Age—29–63 years old and 32–65 years old Sex—There were 19 men and 15 women in the control group. There were 18 men and 16 women in the experimental group Ethnicity—Not stated	Age—Average age 35.5 Sex—2 male 2 female Ethnicity—Not stated	Age—No mean stated but ages ranged from 18 to 54 Sex—17 males and 12 females Ethnicity—Not stated
Statistical analysis performed	Not listed	SPSS	Student *t*‐tests	*t* tests and Data was analyzed using Microsoft Excel 2013 and Graphpad prism version 8

### 2.2 Data Items

All outcomes sought were related to metabolic parameters that olanzapine has been shown to affect: glucose levels, lipid profile (which included triglycerides HDL, LDL and total cholesterol), weight gain, and waist circumference.

### 2.3 Study Risk of Bias Assessment

Bias assessment and overall analysis within the selected studies was performed using the ROBINS I tool [21] for the open label trials and the CASP (Critical Appraisal Skill Programme) tool [22] for the RCTs. The JBI (Joanna Briggs Institute) tool [23] was used for the case series included.

### 2.4 Effect Measures

The effect measures of outcomes were included in the presentation of results as seen in Tables 3 and 4

### 2.5 Synthesis Method

The data from the included studies was inserted into Tables 3 and 4. Key outcomes and data were assessed and compared between studies. The data was then reviewed for the most appropriate method of synthesis. Heterogeneity was assessed by evaluating the individual papers and considering multiple factors such as study design, patient demographics, sample size, dose of intervention given, variation in statistical analysis, and metabolic parameters measured as seen in Table 3. Given the heterogeneity of the selected studies, a narrative synthesis was performed using the extracted data from Tables 3 and 4.

### 2.6 Reporting Bias

To minimize any bias due to missing results in the synthesis, a comprehensive search strategy was formed. The search strategy formed had a clear outline of the inclusion and exclusion criteria to ensure a broad range of results was produced from multiple search engines.

### 2.7 Certainty Assessment

This was carried out within the risk of bias assessments within the CASP, ROBINS I and JBI tools. These tools assessed study design, sample size, appropriateness, evaluated the risk of bias, assessed for patterns across the studies, and the overall coherence and quality of the study (Supporting Information).

## 3 Results

Key outcomes were extracted from all the 7 papers and analyzed thoroughly. The key outcomes in this analysis were the effects adjunctive aripiprazole had on weight/BMI, waist circumference, fasting triglycerides, fasting cholesterol (total, HDL, LDL), fasting glucose, and psychiatric control using the positive and negative symptom scale score (PANSS score). A more detailed table of results can be seen in Tables 3 and 4.

### 3.1 Effect of Adjunctive Aripiprazole on Weight and BMI

The addition of aripiprazole to olanzapine treatment demonstrated broadly positive effects on weight and BMI across studies. Henderson et al. [8] reported a statistically significant weight reduction (*p* = 0.003) of 1.3 kg and a BMI decrease of 0.4 kg/m^2^ in the aripiprazole group. Khaleel et al. [24] recorded a modest but statistically significant BMI decrease of 0.2 kg/m^2^ (*p* = 0.000). Englisch et al. [25] reported a statistically significant decrease in both weight (*p* < 0.05) and BMI (*p* < 0.05) with a decrease of 0.7 kg and 0.2 kg/m^2^ respectively. However, Gupta and colleagues [9] found no statistically significant weight loss or BMI reduction, indicating variability in patient responses. In fact, the study found an increase in weight of 0.43 kg. However, Wang et al. [26] showed a more pronounced weight loss of 2.2 kg and a BMI reduction of 0.8 kg/m^2^, but this was not statistically significant. The other study conducted by Sulejmanpasic et al. [27] did not provide precise weight or BMI data, although it did claim statistically significant decreases in both weight and BMI.

### 3.2 Effect on Waist Circumference

Limited studies reported changes in waist circumference. Henderson et al. [8] observed a slight reduction in waist circumference by 0.76 cm that did not reach statistical significance. Whereas Gupta et al. [9] found a small increase in waist circumference by 0.73 cm, suggesting that aripiprazole may not consistently impact central adiposity. Other studies did not record waist circumference changes.

### 3.3 Effect on Fasting Triglycerides

A significant reduction in fasting triglycerides was observed in multiple studies. Henderson et al. [8] found a statistically significant decrease (*p* = 0.001) of 51.7 mg/dL. Gupta et al. [9] also noted a statistically significant reduction (*p* = 0.001) of triglyceride levels. While Wang et al. [26] reported a more substantial 74.0 mg/dL decrease which did meet statistical significance (*p* = 0.006). Jia et al. [28] used bar graphs without exact numerical data, making comparisons difficult, but the bar graphs suggested a slight nonstatistically significant decrease in triglyceride levels. Sulejmanpasic et al. [27] did not provide precise data on triglyceride levels; although, it did claim a statistically significant decrease. Other studies did not record triglyceride levels. These findings suggest that aripiprazole may help mitigate olanzapine‐induced hypertriglyceridemia.

### 3.4 Effect on Cholesterol (Total, HDL, and LDL)

Results of cholesterol levels were inconsistent across studies. Henderson et al. [8] found no significant reductions in total cholesterol (−3 mg/dL), HDL (+0.4 mg/dL), or LDL (LDL −0.2 mg/dL). Gupta et al. [9] observed similar trends with a non‐significant reduction in total cholesterol and a slight HDL and LDL increase. Jia et al. [28] used bar graphs without exact numerical data, making comparisons difficult, but the bar graphs suggested a slight, non‐statistically significant decrease in total cholesterol. Sulejmanpasic et al. [27] did not provide precise data on cholesterol levels; although, it did claim a statistically significant decrease in total cholesterol. Other studies did not measure the effect of adjunctive aripiprazole on cholesterol levels.

### 3.5 Effect on Fasting Glucose

The impact of aripiprazole on fasting glucose was varied. Henderson et al. [8] observed a non‐significant decrease (−2 mg/dL), while Gupta et al. [9] reported a slight increase. Jia et al. [28] used bar graphs without exact numerical data, suggesting a slight, non‐statistically significant decrease in fasting glucose. Khaleel et al. [24] showed a substantial statistically significant decrease of 6.7 mg/dL, supporting potential metabolic benefits. Sulejmanpasic et al. [27] did not provide precise data on fasting glucose levels; although it did claim a statistically significant decrease in fasting glucose.

### 3.6 Effect on Psychiatric Symptom Control (PANSS Score)

The positive and negative symptom scale score (PANSS score), which measures psychiatric symptom severity, remained stable in all studies that measured it. Henderson et al. [8] found no significant difference between groups. Gupta et al. [9] reported a slight, non‐statistically significant increase in the PANSS score (+1). Both Sulejmanpasic et al. [27] and Englisch et al. [25] provided no specific data but claimed no statistically significant changes in the PANSS score. Jia et al. [28] found a significant improvement after initiating treatment, though their population consisted of first‐time schizophrenia patients. Khaleel et al. [24] did not measure the PANSS score, and Wang et al. [26] did not provide data for the olanzapine group in isolation.

## 4 Discussion

### 4.1 Summary of Key Findings

Based on the studies reviewed, adjunctive aripiprazole shows positive but varied weight loss benefits, with Henderson et al. [8], Wang et al. [26], Sulejmanpasic et al. [27], Englisch et al. [25] and Khaleel et al. [24] reporting significant reductions in weight and BMI, while Gupta et al. [9] found no meaningful change. Statistically significant decreases in fasting triglycerides were consistent across multiple studies, including studies by Henderson et al. [8], Gupta et al. [9], Wang et al. [26], and Sulejmanpasic et al. [27]. This supports the hypothesis that aripiprazole may counteract some of the metabolic adverse effects of olanzapine. Effects on cholesterol and fasting glucose were less positive, with some studies, specifically Henderson et al. [8], Gupta et al. [9], Jia et al. [28] and Englisch et al. [25] showing nonstatistically significant reductions. Finally, psychiatric symptom control remained stable in all 7 studies included in this systematic review, suggesting that aripiprazole does not negatively affect schizophrenia symptoms while potentially providing metabolic advantages.

### 4.2 Interpretation of Findings

A consistent finding among the study results was the effect of adjunctive aripiprazole on serum fasting triglyceride levels in patients receiving olanzapine treatment. Upon reviewing the studies selected in this systematic review, there is a consistency in the reduction of fasting triglyceride levels as seen in Henderson et al. [8], Gupta et al. [9], Wang et al. [26], and Sulejmanpasic et al. [27]. However, it must be noted that not all studies showed the reduction to be statistically significant, as demonstrated with the study by Jia et al. [28]. Nonetheless, the overall trend is consistent with prior randomized controlled trials, such as Henderson et al. [8] and findings from the broader background literature such as Gupta et al. [9], both of which reported statistically significant reductions in serum fasting triglyceride levels. These results suggest that our findings are in alignment with existing evidence supporting the efficacy of adjunctive aripiprazole in lipid profile improvement. It must be appreciated that hypertriglyceridemia is thought to be an independent risk factor for cardiovascular disease [29] therefore, it has been suggested that a reduction in serum triglyceride levels would reduce cardiovascular morbidity [26]. The exact mechanism of reducing triglyceride levels in these patients is unknown.

The result suggests that adjunctive aripiprazole may contribute to weight reduction in patients treated with olanzapine, though the effect varies across studies. Several studies [8, 24, 25, 26, 27] reported statistically significant reductions in both body weight and BMI, indicating a potential for aripiprazole to counteract antipsychotic‐induced weight gain. However, this effect was not consistent in all cases; for instance, in a larger open‐label trial by Gupta et al. [9] a slight weight increase was observed, possibly due to the lower dose of aripiprazole used (5 mg daily). This contrasts with most other studies, which employed doses of 10 mg or higher.

Despite the observed range in dosing (5–15 mg/day), a clear dose‐dependent relationship has not been established. While some studies using higher doses reported metabolic improvements, others did not show consistent benefits at either end of the dosage spectrum. This lack of a clear dose–response may be attributable to heterogeneity in study design, participant characteristics, duration of treatment, and the specific metabolic outcomes measured. Furthermore, while reductions in BMI and weight were often statistically significant, their clinical relevance in terms of overall metabolic health remains uncertain. Overall, the findings suggest a potential role for adjunctive aripiprazole in mitigating olanzapine‐induced weight gain, but further dose‐ranging and long‐term studies are needed to clarify the optimal therapeutic dose and to determine whether these effects translate into meaningful health benefits.

The findings on cholesterol values suggest that adjunctive aripiprazole does not have a meaningful impact on improving these metabolic markers. Across multiple datasets, specifically, Gupta et al. [9], Englisch et al. [25] and Jia et al. [28], changes in total cholesterol, HDL, and LDL cholesterol were small and lacked statistical significance. The lack of a statistically significant trend across different studies suggests that adjunctive aripiprazole does not lead to cholesterol improvement in patients taking antipsychotics like olanzapine. This is consistent with the findings from the RCT by Henderson et al. [8].

Similarly, the impact of adjunctive aripiprazole on glucose regulation appears negligible, and this is inconsistent with the findings from the RCT by Henderson et al. [8]. The evidence does not support the use of adjunctive aripiprazole as an intervention for glucose regulation in patients receiving olanzapine. As mentioned previously, its role appears to be limited to addressing weight gain, as suggested by Cooper et al. [16]. Reported changes in fasting glucose levels were inconsistent, with Khaleel et al. [24] and Sulejmanpasic et al. [27] indicating a statistically significant decrease, while Henderson et al. [8], Gupta et al. [9], Jia et al. [28] and Englisch et al. [25] showed no meaningful difference. The lack of statistical significance in these findings supports the conclusion that aripiprazole does not provide significant metabolic benefits in terms of glucose control.

However, one consistent finding across all studies reviewed in this systematic review was that the psychiatric control scores remained consistent. This suggests that an aripiprazole adjunct will not cause worsening of psychiatric symptoms. This is consistent with the findings from the RCT by Henderson et al. [8], which showed no statistically significant differences in PANSS scores between groups with and without adjunctive aripiprazole.

While several studies in this review report statistically significant changes in metabolic parameters, particularly weight, BMI, and fasting triglyceride levels, it remains unclear whether these changes translate to clinically meaningful improvements in cardiovascular risk or reduction in metabolic syndrome. For instance, Henderson et al. [8] reported a 1.3 kg weight loss and a 0.4 kg/m^2^ reduction in BMI (*p* = 0.003). Similarly, Gupta et al. [9] and Wang et al. [26] observed statistically significant reductions in fasting triglycerides. However, in clinical terms, guidelines suggest that reductions in triglycerides of at least 0.5 mmol/L (≈44.2 mg/dL) are needed to impact cardiovascular outcomes [29], a threshold only narrowly met or exceeded in some studies. Moreover, reductions in BMI of less than 1 kg/m^2^ are unlikely to produce substantial long‐term cardiovascular benefit or reduce morbidity associated with metabolic syndrome. Importantly, most changes in fasting glucose, total cholesterol, and LDL/HDL levels were not statistically significant and fell short of thresholds considered clinically significant. Therefore, while adjunctive aripiprazole appears to have a statistically significant impact on some metabolic markers, particularly triglycerides, the clinical significance of these changes, especially in terms of long‐term cardiovascular risk reduction, remains questionable and requires further investigation in larger, longer‐term trials.

While current established clinical guidelines, such as those issued by the National Institute for Health and Care Excellence (NICE) in the UK [30] or the American Psychiatric Association (APA) in the US [31], provide comprehensive recommendations for monitoring and managing metabolic adverse effects associated with antipsychotics, a universal endorsement for adjunctive aripiprazole specifically to mitigate olanzapine‐induced metabolic dysfunction is not yet a standard recommendation. These guidelines primarily advocate for routine metabolic monitoring, lifestyle interventions, considering a switch to antipsychotics with lower metabolic risk profiles, or the use of agents like metformin.

This systematic review contributes to the evolving understanding of managing antipsychotic‐induced metabolic side effects by synthesizing the available evidence on adjunctive aripiprazole. The findings herein, derived from various study designs including randomized controlled trials, demonstrate the potential efficacy of this approach in improving certain metabolic parameters. While this evidence is valuable, the ultimate integration of adjunctive aripiprazole into widely accepted clinical guidelines would necessitate further large‐scale, methodologically robust studies to confirm long‐term safety, effectiveness, and cost‐effectiveness. This review therefore serves to consolidate the current evidence base, highlighting an important area for future research and clinical consideration.

### 4.3 Limitations

#### 4.3.1 Confounding

In the included studies, confounding variables are not controlled consistently. Potential confounders such as diet and exercise, although controlled in some of the studies, are not explicitly stated to be controlled in all. These confounders can potentially affect all aspects of metabolic health such as weight and lipid levels. Patients who implement positive lifestyle changes such as increasing the amount of exercise they perform and reducing calorific intake from their diet would possibly see changes in their metabolic parameters regardless of adjunctive aripiprazole.

#### 4.3.2 Study Sizes

The studies included in this systematic review varied widely in sample size, ranging from as few as 4 participants to as many as 68. Smaller studies, such as those with sample sizes of 4 or 8, are inherently more susceptible to random error and may lack the statistical power to detect true clinical effects. This can lead to overestimation or underestimation of treatment outcomes and limits the generalizability of findings. Although these smaller studies may be less reliable from a statistical standpoint, they still offer valuable insights into the potential metabolic effects of adjunctive aripiprazole. Their inclusion contributes to a more comprehensive synthesis of the available literature but also adds to the heterogeneity observed across studies, which may affect the robustness of pooled conclusions. Due to the diversity in study design and outcome reporting, formal sensitivity analyses based on sample size were not feasible. However, the sample size of each study was acknowledged in the descriptive synthesis and considered when interpreting the overall evidence. Large‐scale trials are necessary to provide more definitive conclusions regarding the efficacy and safety of adjunctive aripiprazole in mitigating olanzapine‐associated metabolic adverse effects.

#### 4.3.3 Study Duration

Studies showed variation in duration ranges from 8 to 12 weeks. Longer studies would be more insightful as they can assess the effect of adjunctive aripiprazole on the metabolic health of patients long term and can see if the metabolic benefit translates to reduced cardiovascular mortality and life expectancy. Longer study duration would not only be beneficial in assessing the efficacy of adjunctive aripiprazole, but it would also assess longer‐term side effects on patients and see how psychiatric symptom control is affected long term.

#### 4.3.4 Methodology

The analysis in this systematic review was a narrative synthesis due to the heterogeneity in the included studies. As a result, this systematic review lacks the statistical robustness of a meta‐analysis. On top of this, statistical representation of data was difficult to extract from certain studies; for example, Jia et al. [28] represented data in bar graphs rather than tables. Another study [27] did not include its precise data at all. In addition to this, study designs varied and included some studies from the lower end of the pyramid of hierarchical evidence, which impacts the certainty of the analysis [32]. The risk of bias appeared low within the randomized control trials, open‐label trials, and the case series, details of which can be seen in the Supporting Information.

Overall, a narrative synthesis of the studies revealed some statistically significant improvements in triglyceride levels and variable effects on weight loss. While the long‐term impact on metabolic health and potential reduction in cardiovascular‐related mortality and morbidity remain uncertain, the addition of aripiprazole did not negatively affect patients' psychiatric outcomes.

## 5 Recommendations

To enhance the clinical utility of adjunctive aripiprazole in managing olanzapine‐induced metabolic adverse effects, future research should focus on conducting well‐powered, large‐scale randomized controlled trials that address the limitations of previous studies. These trials should include diverse populations across age, sex, and ethnicity, and be conducted over extended durations, ideally from months to years, to monitor both beneficial and adverse effects comprehensively. It is essential to control for relevant confounders such as diet, physical activity, and concurrent use of lipid‐lowering medications like statins. Future studies should aim not only to confirm the effectiveness of adjunctive aripiprazole but also to determine optimal dosing regimens and treatment durations that maximize metabolic benefits without compromising psychiatric stability. A broader evaluation of metabolic outcomes including insulin resistance, adipokines, and inflammatory markers such as C‐reactive protein (CRP) and interleukin‐6 (IL‐6) would provide a more comprehensive understanding of its systemic effects. Stratifying participants based on baseline metabolic risk (e.g., obesity, dyslipidaemia, or prediabetes) could help identify subgroups most likely to benefit. Additionally, mechanistic studies are needed to elucidate the pharmacodynamic and molecular pathways underlying aripiprazole's metabolic effects, distinguishing direct drug actions from secondary behavioral or hormonal changes. Addressing these areas will inform evidence‐based clinical guidelines and support personalized treatment strategies for patients with schizophrenia at risk of metabolic complications.

## 6 Summary and Conclusion

This systematic review carried out an investigation to determine if adjunctive aripiprazole is effective in reducing olanzapine‐induced metabolic adverse effects in patients with schizophrenia. Specific metrics were used to assess the effects adjunctive aripiprazole had on metabolic health, such as glucose levels, lipid profile, bodyweight/BMI, PANSS score, and waist circumference. This systematic review analyzed and narratively synthesized the results from a variety of different research designs, including randomized control trials, open‐label trials, and case series. This broad range of different study designs is likely due to a lack of current evidence on the specific research question of this study. Key findings of the studies reviewed showed adjunctive aripiprazole resulted in a statistically significant decrease in fasting triglycerides, which was consistent across studies, but not a statistically significant decrease in other lipid levels, such as cholesterol. The studies highlighted some potential weight loss benefits, with some studies reporting significant reductions in weight and BMI, while one other found no meaningful change. However, this may be a dose‐dependent outcome, as the study that found no significant change in weight used a substantially lower dose of aripiprazole compared to other studies. Importantly, psychiatric symptom control remained stable in most studies, demonstrating that aripiprazole does not negatively affect psychotic symptoms while potentially providing metabolic advantages.

In conclusion, this review acknowledges that adjunctive aripiprazole may reduce some of the metabolic adverse effects of olanzapine (hypertriglyceridemia and weight gain), but RCT‐controlled trials are warranted to further assess the efficacy, effective dose, and long‐term metabolic benefit of the addition of aripiprazole. Further evidence would be necessary before recommending prescribing aripiprazole in combination with olanzapine in a clinical setting.

## Author Contributions

Stephen Simmons read all articles and designed the PRISMA flow chart, analyzed relevant articles, wrote the whole manuscript, and checked references. Soban Sadiq designed the project, wrote the title, reviewed all articles, and reviewed the analysis and the whole manuscript.

## Disclosure

Registry and the registration no. of the systematic review study: PROSPERO registration ID CRD420251057301.

## Ethics Statement

The authors have nothing to report.

## Consent

The authors have nothing to report.

## Conflicts of Interest

The authors declare no conflicts of interest.

## Supporting information


**Data S1:** npr270046‐sup‐0001‐Supinfo1.docx.

## Data Availability

Data is available as a Supporting Information in the public repository figshare DOI: https://doi.org/10.6084/m9.figshare.29581148.
