# Supplementary material for: The Impact of Adjunctive Aripiprazole on Olanzapine‐Induced Metabolic Adverse Effects in Patients With Schizophrenia: A Systematic Review
Source: Neuropsychopharmacol Rep. 2025 Aug 31;45(3):e70046. doi: 10.1002/npr2.70046 (PMC12399328; doi:10.1002/npr2.70046)
Supplement: Supplementary file 1 — Data S1: npr270046‐sup‐0001‐Supinfo1.docx. [file NPR2-45-e70046-s001.docx]

**PRISMA Checklist**


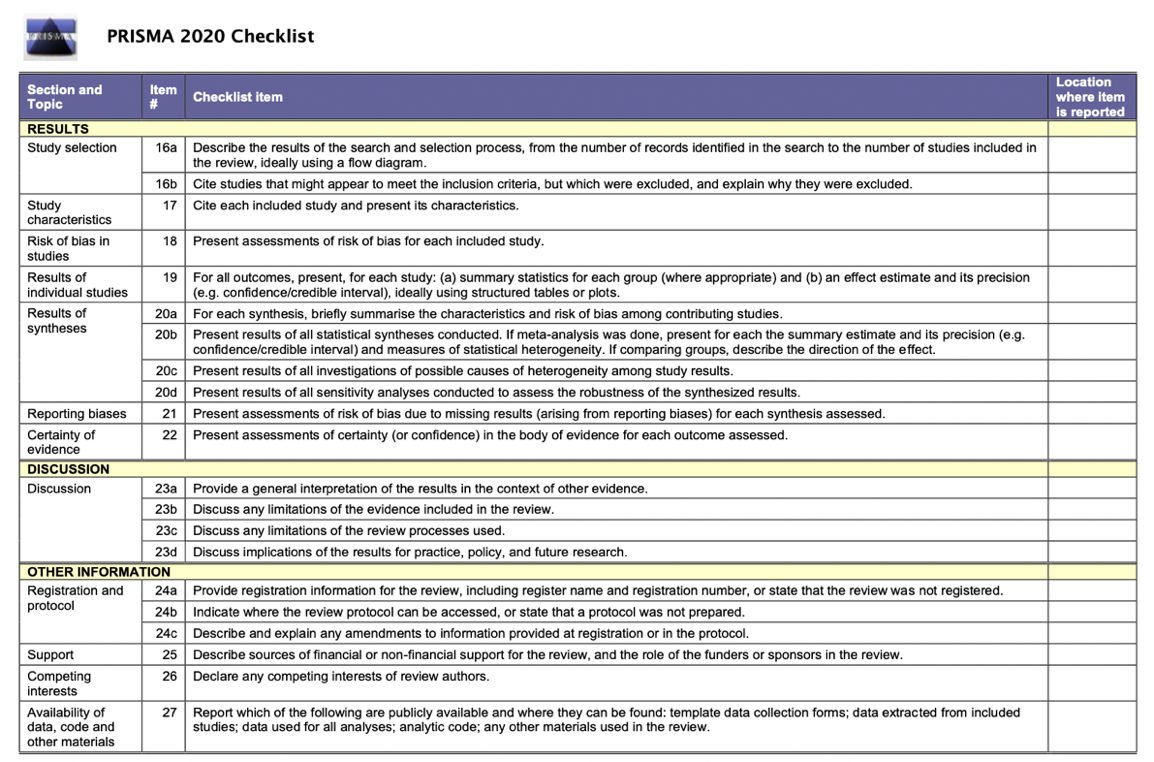

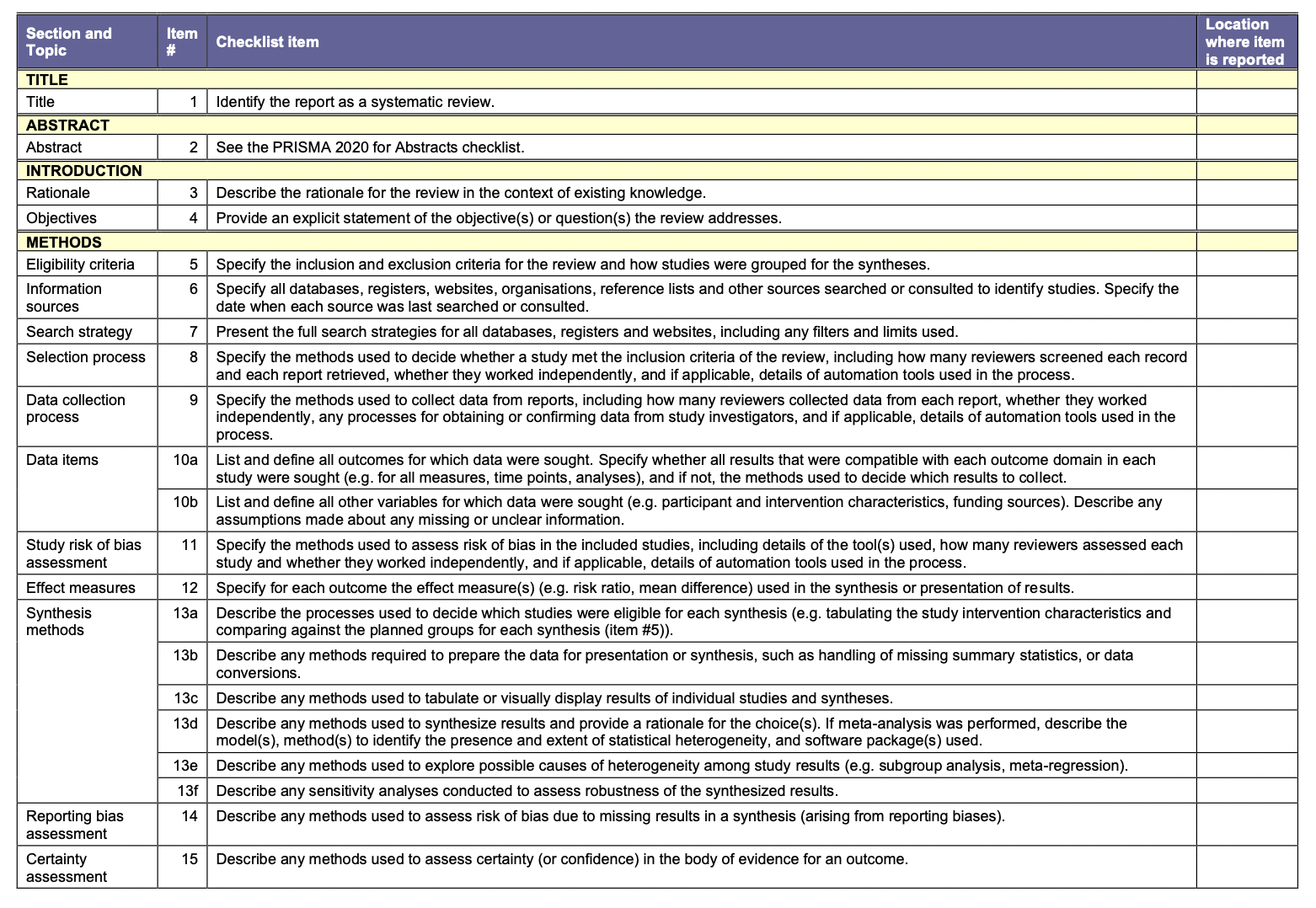


**Search Strategy**

| **Description of search** | **Search details** |
| --- | --- |
| Identify the key words for your search based on the PICO analysis (1st term refers to population, 2^nd^ term  to intervention, 3^rd^  term to outcome)    These key words are the pillars of your search, each search must contain these key words. | #1 Olanzapine  #2 Aripiprazole adjunct  #3 Metabolic adverse effects  #4 Weight gain  #5 Schizophrenia |
| Combine these key words with the Boolean operator  “AND” | Long string format  Olanzapine  AND Aripiprazole adjunct AND Metabolic adverse effects AND Weight gain AND Schizophrenia  Line format  #1 Olanzapine  #2 Aripiprazole adjunct  #3 Metabolic adverse effects  #4 Weight gain  #5 Schizophrenia  #6 #1 AND #2 AND #3 AND #4 AND #5 |
| Search for synonyms and similar words | Olanzapine  Zyprexa  Aripiprazole adjunct  Aripiprazole  Abilify adjunct  Abilify addition  Aripiprazole addition  Metabolic adverse effects  Metabolic side effects  Weight gain  Weight increase  BMI increase  Schizophrenia  Psychotic disorder  Psychosis |
| Add the synonyms to the key words by using the Boolean Operator “OR” | Long string format  (Olanzapine OR Zyprexa) AND  (Aripiprazole adjunct OR Aripiprazole OR Abilify adjunct OR Abilify addition OR Aripiprazole addition) AND (Metabolic adverse effects OR Metabolic side effects) AND (Weight gain OR Weight increase OR BMI increase) AND (Schizophrenia OR Psychotic disorder OR Psychosis)  Line format  #1 Olanzapine OR Zyprexa  #2 Aripiprazole adjunct OR Aripiprazole OR Abilify adjunct OR Abilify addition OR Aripiprazole addition  #3 Metabolic adverse effects OR Metabolic side effects  #4 Weight gain OR Weight increase OR BMI increase  #5 Schizophrenia OR Psychotic disorder OR Psychosis  #6 #1 AND #2 AND #3 AND #4 AND #5 |
| Additional technique used | Snowballing technique used |

| **Final search terms used and number of results retrieved** | **Google scholar**  #1 Olanzapine OR Zyprexa  #2 Aripiprazole adjunct OR Aripiprazole OR Abilify adjunct OR Abilify addition OR Aripiprazole addition  #3 Metabolic adverse effects OR Metabolic side effects  #4 Weight gain OR Weight increase OR BMI increase  #5 Schizophrenia OR Psychotic disorder OR psychosis  #6 #1 AND #2 AND #3 AND #4 AND #5  239 results |
| --- | --- |
|  | **Pubmed/Medline**  #1 Olanzapine OR Zyprexa  #2 Aripiprazole adjunct OR Aripiprazole OR Abilify adjunct OR Abilify addition OR Aripiprazole addition 5375  #3 Metabolic adverse effects OR Metabolic side effects  #4 Weight gain OR Weight increase OR BMI increase  #5 Schizophrenia OR Psychotic disorder  #6 #1 AND #2 AND #3 AND #4 AND #5  85 results |
|  | **Embase**  #1 Olanzapine OR Zyprexa  #2 Aripriprazole adjunct OR Aripiprazole OR Abilify adjunct OR Abilify addition OR Aripriprazole addition  #3 Metabolic adverse effects OR Metabolic side effects  #4 Weight gain OR Weight increase OR BMI increase  #5 Schizophrenia OR Psychotic disorder  #6 #1 AND #2 AND #3 AND #4 AND #5  459 results |
|  | **Psychinfo**  #1 Olanzapine OR Zyprexa  #2 Aripiprazole adjunct OR Aripiprazole OR Abilify adjunct OR Abilify addition OR Aripiprazole addition  #3 Metabolic adverse effects OR Metabolic side effects  #4 Weight gain OR Weight increase OR BMI increase  #5 Schizophrenia OR Psychotic disorder OR psychosis  #6 #1 AND #2 AND #3 AND #4 AND #5  32 results |
|  | **Cochrane library**  #1 Olanzapine OR Zyprexa  #2 Aripiprazole adjunct OR Aripiprazole OR Abilify adjunct OR Abilify addition OR Aripiprazole addition  #3 Metabolic adverse effects OR Metabolic side effects  #4 Weight gain OR Weight increase OR BMI increase  #5 Schizophrenia OR Psychotic disorder OR psychosis  #6 #1 AND #2 AND #3 AND #4 AND #5  32results |
| **Inclusion criteria** | Articles published in the last 20 years  Articles published in the English language  Studies with adult schizophrenia patients  Quantitative studies |
| **Exclusion criteria** | Qualitative studies  Articles including non adult studies  Animal studies |

**Critical appraisal of Study 1 using CASP tool**

| **Study design 1** |  |
| --- | --- |
| **Did the study address a clearly focused research question?** | Yes. The study focused on the effect aripiprazole has When added to Overweight and Obese Olanzapine-treated Schizophrenia Patients. Specifically looking at the metabolic effects. A physical examination and medical history were performed at baseline and measurements of vital signs, weight, height, body mass index (BMI) as well as waist (supra-iliac) and hip circumference were performed at each visit. Several metabolic markers were measured, such as LDL, HDL, VLDL-cholesterol, triglycerides, intermediate-density lipoprotein (IDL-c), and lipoprotein(a). |
| **Was the assignment of participants to interventions randomised?** | Yes. The study is a double blind and consisted of two random order 4-week treatment arms (aripiprazole 15 mg or placebo) separated by a 2-week adjunctive treatment washout. |
| **Were all participants who entered the study accounted for at its conclusion?** | Yes, 16 patients consented for the study. However, one patient was found to be ineligible for the study due to recent changes in medication, and one patient dropped out of the study due to social circumstances. |
| **Study methodology** |  |
| - **Were the participants ‘blind’ to intervention they were given?** - **Were the investigators ‘blind’ to the intervention they were giving to participants?** - **Were the people assessing/analysing outcome/s ‘blinded’?** | Yes  Yes  Yes |
| **Were the study groups similar at the start of the randomised control trial?** | As the trial consists of 2 arms The 2 study groups receive both the placebo and the intervention. The mean age of subjects was 49±8 years and ten (67%) were male. Three subjects were African American (20%) and twelve (80%) were Caucasian. |
| **Apart from the experimental intervention, did each study group receive the same level of care (that is, were they treated equally)?** | No, the participants remained being treated for their differing co-morbidities. Three were treated for hyperlipidaemia with a lipid-lowering statin; three were treated for hypertension (with a beta-blocker); one for hypothyroidism (with thyroid replacement); and one for type 2 DM (with metformin). |
| **Results** |  |
| **Were the effects of intervention reported comprehensively?** | Yes. The data was analysed using SPSS (version 13.0; SPSS inc., Chicago, IL). Many metabolic changes were measured such as BMI, weight and lipid levels. P values were included. |
| **Was the precession of the estimate of the intervention or treatment effect reported?** | No confidence interval reported |
| **Do the benefits of the experimental intervention outweigh the harms and costs?** | Can’t tell. The combination of olanzapine and aripiprazole cost may be too high and alternatives approaches such as switching antipsychotic medication may be more suitable. |
| **Will the results help locally.** |  |
| **Can the results be applied to your local population?** | Can't tell, don’t know if psychotic patients locally are similar to those in the study. |
| **Would the experimental intervention provide greater value to the people in your care than any of the existing interventions ?** | Can’t tell, as long-term, placebo-controlled trials are warranted to further assess the efficacy, safety, cost effectiveness, and metabolic benefit of the addition of aripiprazole to olanzapine-treated patients. |

**Critical Appraisal of study 2 and study 3 using ROBINS I tool**

| Bias in selection of participants into the study | 1.1. Was selection of participants into the study (or into the analysis) based on participant characteristics observed after the start of intervention? **If N/PN to 1.1:** go to 1.4 | Paper 2: No. The participants were selected before the intervention was given. The study recruited outpatients aged 21–65 years with a diagnosis of schizophrenia. The patients must have been on a stable dose of olanzapine, clozapine, or risperidone, for at least 1 month and be suffering from metabolic adverse effects. The study excluded subjects who had a previous allergy to aripiprazole. Participants with current substance misuse or those non-adherent to current prescribed medications were excluded. Participants who had clinically significant abnormalities requiring medications such as lipid lowering agents or antidiabetic medication, were also excluded. Women who are pregnant, breast feeding, or planning to conceive were also excluded.  Paper 3: No. The participants were selected before the intervention was given. Patients were aged between 18 and 65 years with a diagnosis of schizophrenia. The patients must have been on a stable dose of olanzapine, clozapine, or risperidone, for at least 1 month and be suffering from metabolic abnormalities (including being overweight, obesity, hyperglycaemia, or dyslipidaemia). Women who are pregnant, breast feeding, or planning to conceive were excluded. |
| --- | --- | --- |
|  | 1.2. **If Y/PY to 1.1**: Were the postintervention variables that influenced selection likely to be associated with intervention?  1.3 **If Y/PY to 1.2**: Were the postintervention variables that influenced selection likely to be influenced by the outcome or a cause of the outcome? | N/A  N/A |
|  | 1.4. Do start of follow-up and start of intervention coincide for most participants? | Paper 2: Yes. The participants were followed up and monitored directly after the intervention was given.  Paper 3: Yes. The participants were followed up and monitored directly after the intervention was given. |
|  | 1.5. **If Y/PY to 1.2 and 1.3, or N/PN to 1.4**: Were adjustment techniques used that are likely to correct for the presence of selection biases? | NA |
|  | **Risk of bias judgement** | Paper 2: Low  Paper 3: Low |
|  |  |  |

| Bias in classification of  interventions | 2.1 Were intervention groups clearly defined? | Paper 2:Yes  As this is a single arm open label study all subjects received the intervention.  Paper 3:Yes  As this is a single arm open label study all subjects received the intervention. |
| --- | --- | --- |
|  | 2.2 Was the information used to define intervention groups recorded at the start of the intervention? | Paper 2:Yes  Paper 3:Yes |
|  | 2.3 Could classification of intervention status have been affected by knowledge of the outcome or risk of the outcome? | Paper 2:No  As there was one group, all patients in the study were given the intervention.  Paper 3:No  As there was one group, all patients in the study were given the intervention. |
|  | **Risk of bias judgement** | Paper 2 : Low  Paper 3: low |
|  |  |  |

| Bias due to deviations from intended interventions |  |  |
| --- | --- | --- |
|  | 3.3 Were important co-interventions balanced across intervention groups? | Paper 2: Yes Participants who had clinically significant abnormalities on enrolment examination and screening that required active intervention, that is, initiation of lipid lowering agent or antidiabetic medication, were excluded.  Paper 3: No  Patients were maintained on their current dose of all medicines. This may include lipid lowering agents or anti diabetic medication, although this is not explicitly stated. |
|  | 3.4. Was the intervention implemented successfully for most participants? | Paper 2: Yes  The intervention was implemented as intended in most participants.  Paper 3: Yes  The intervention was implemented as intended in most participants. |
|  |  |  |
|  | 3.5. Did study participants adhere to the assigned intervention regimen? | Paper 2: Yes  Out of the 67 initial participants, 1 was withdrawn from the study due to non compliance. Note patients who were non compliant to their current medications were excluded from being inducted to the study.  Paper 3: Yes  Out of the 49 participants, none were withdrawn due to non compliance. |
|  | 3.6. If N/PN to 4.3, 4.4 or 4.5: Was an appropriate analysis used to measure the effect of starting and adhering to the intervention? | Paper 2: N/A  Paper 3: Yes  Body weight, BMI, plasma levels of fasting glucose, triglycerides, total cholesterol, HDL-C, LDL-C, and adiponectin were measured at both baseline and week 8. |
|  | Risk of bias judgment | Paper 2: Low  Paper 3: Low |

| Bias due to missing data | 4.1 Were outcome data available for all, or nearly all, participants? | Paper 2:Yes  Of the 67 initial participants Outcome data is available for 55. Four subjects dropped out of the study: three due to adverse effects and one was lost to follow-up. Eight participants were withdrawn from the study: four due to patient/family request, two due to social issues (housing/work), one due to deterioration in mental state assessed to be unrelated to the study medication and one due to non-compliance.  Paper 3: Yes  Of the 49 initial patients, outcome data is available for 43. 3 withdrew consent and 3 were lost during follow-up. |
| --- | --- | --- |
|  | 4.2 Were participants excluded due to missing data on intervention status? | Paper 2: No  As stated previously Eight participants were withdrawn from the study: four due to patient/family request, two due to social issues (housing/work), one due to deterioration in mental state assessed to be unrelated to the study medication and one due to non-compliance.  Paper 3: No  No patients were withdrawn/eliminated from the study by the researchers. |
|  | 4.3 Were participants excluded due to missing data on other variables needed for the analysis? | Paper 2: No  Paper 3: No |
|  | 4.4 **If PN/N to 5.1, or Y/PY to 5.2 or 5.3**: Are the proportion of participants and reasons for missing data similar across interventions? | Paper 2:N/A  Paper 3:N/A |
|  | 4.5 **If PN/N to 5.1, or Y/PY to 5.2 or 5.3**: Is there evidence that results were robust to the presence of missing data? | Paper 2:N/A  Paper 3:N/A |
|  | **Risk of bias judgement** | Paper 2: Low  Paper 3:Low |
|  |  |  |

| Bias in  measurement of outcomes | 5.1 Could the outcome measure have been influenced by knowledge of the intervention received? | Paper 2: No  Outcomes measured were quantitative Weight, BMI and waist circumference were measured. Serum level of total cholesterol, HDL, LDL, and triglycerides and glucose levels were also measured. Assessor judgment is therefore negligible and risk of bias low.  Paper 3: No Outcomes measured were quantitative.Body weight, BMI, plasma levels of fasting glucose, triglycerides, total cholesterol, HDL-C, LDL-C, and adiponectin were measured. Assessor judgment is therefore negligible and risk of bias low |
| --- | --- | --- |
|  | 5.2 Were outcome assessors aware of the intervention received by study participants? | Paper 2: Yes  As this is an open label study The outcome assessors were aware of the intervention given.  Paper 3: Yes  As this is an open label study The outcome assessors were aware of the intervention given. |
|  | 5.3 Were the methods of outcome assessment comparable across intervention groups? | Paper 2:N/A as there is only one intervention group.  Paper 3: N/A as there is only one intervention group. |
|  | 5.4 Were any systematic errors in measurement of the outcome related to intervention received? | Paper 2: No  Paper 3: No |
|  | **Risk of bias judgement** | Paper 2: Low  Paper 3: Low |
|  |  |  |

| Bias in selection of the reported result | Is the reported effect measurement likely to be selected, on the basis of the results, from...  6.1. ... multiple outcome *measurements* within the outcome domain? | Paper 2: No  All measurements stated are reported. Therefore there is no risk so selective reporting on the basis of the results.  Paper 3: No  All measurements stated are reported.  Therefore there is no risk so selective reporting on the basis of the results. |
| --- | --- | --- |
|  | 6.2 ... multiple *analyses* of the intervention outcome relationship? | Paper 2: No  Multiple analyses weren’t not carried out to generate multiple measures of the effect of the intervention.  Paper 3:No  Multiple analyses weren’t not carried out to generate multiple measures of the effect of the intervention. |
|  | 6.3 ... different *subgroups*? | Paper 2: No  There are no subgroups present in this study.  Paper 3:No  There are no subgroups present in this study. |
|  | **Risk of bias judgement** | Paper 2: Low  Paper 3:Low |
|  |  |  |
| Overall bias | **Risk of bias judgement** | Paper 2: Low  Paper 3: Low |
|  |  |  |
|  |  |  |

**Critical appraisal of study 4 using CASP tool**

| **Study design 4** |  |
| --- | --- |
| **Did the study address a clearly focused research question?** | Yes. The study focused on the effect aripiprazole has When added to Schizophrenia Patients. Specifically looking at weight and BMI as well as assessing tolerability using the PANSS and CGI scoring systems |
| **Was the assignment of participants to interventions randomised?** | Yes. The study is a double blind and consisted of two random order 6-week treatment arms (aripiprazole 15 mg or placebo). |
| **Were all participants who entered the study accounted for at its conclusion?** | Yes, 4 patients consented for the study. 2 received aripiprazole and 2 received placebo. |
| **Study methodology** |  |
| - **Were the participants ‘blind’ to intervention they were given?** - **Were the investigators ‘blind’ to the intervention they were giving to participants?** - **Were the people assessing/analysing outcome/s ‘blinded’?** | Yes  Yes  Yes |
| **Were the study groups similar at the start of the randomised control trial?** | Can't tell, no information provided on participants, for example age ethnicity and metabolic health. |
| **Apart from the experimental intervention, did each study group receive the same level of care (that is, were they treated equally)?** | Can’t tell, not explicitly state if the patients were also being treated with other lipid/weight lowering medication such as statins. |
| **Results** |  |
| **Were the effects of intervention reported comprehensively?** | No. Stated significant reduction in BMI, serum fasting cholesterol triglycerides and glucose but no data provided. |
| **Was the precession of the estimate of the intervention or treatment effect reported?** | No confidence interval reported |
| **Do the benefits of the experimental intervention outweigh the harms and costs?** | Can’t tell. The combination of olanzapine and aripiprazole cost may be too high and alternatives approaches such as switching antipsychotic medication may be more suitable.  Cost analysis was not reported Adverse effects not clearly stated |
| **Will the results help locally.** |  |
| **Can the results be applied to your local population?** | Can't tell, don’t know if psychotic patients locally are similar to those in the study. Larger sample size also required. |
| **Would the experimental intervention provide greater value to the people in your care than any of the existing interventions ?** | Can’t tell, as long-term, placebo-controlled trials are warranted to further assess the efficacy, safety, cost effectiveness, and metabolic benefit of the addition of aripiprazole to olanzapine-treated patients. There are practical considerations to implementing this treatment, such as cost and adverse reactions and clinical efficacy of treatment |

**Critical appraisal of Study 5 using CASP tool**

| **Study design 5** |  |
| --- | --- |
| **Did the study address a clearly focused research question?** | Yes. The study focused on the effect aripiprazole has When added to first episode Schizophrenia Patients. Specifically looking at the lipid levels specifically triglycerides, total cholesterol and glucose levels. |
| **Was the assignment of participants to interventions randomised?** | Yes. The assignment of patients to the experimental group and control group was random, 34 in each. |
| **Were all participants who entered the study accounted for at its conclusion?** | Yes, the 68 patients who consented for the study are accounted for in the conclusion. |
| **Study methodology** |  |
| - **Were the participants ‘blind’ to intervention they were given?** - **Were the investigators ‘blind’ to the intervention they were giving to participants?** - **Were the people assessing/analysing outcome/s ‘blinded’?** | Yes  Can’t tell as not explicitly stated  Can’t tell as not explicitly stated |
| **Were the study groups similar at the start of the randomised control trial?** | Inconclusive. The control group consisted of 19 men and 15 women, with an age range of 29-63 years old with a 3-38 month disease course. The experimental group consisted of 18 men and 16 women, with an age range of 32-65 years old with a disease course of 3-36 months. No mean or standard deviation as provided for age or disease course. No information as provided on the ethnicity of the participants. |
| **Apart from the experimental intervention, did each study group receive the same level of care (that is, were they treated equally)?** | Yes, during the treatment, the two groups of patients were not allowed to use other antidepressants and antipsychotics. Electroconvulsive therapy was not allowed. An ordinary diet was required. High shock and high protein food were not allowed |
| **Results** |  |
| **Were the effects of intervention reported comprehensively?** | Yes. The data was analysed using SPSS2.0. Serum total cholesterol, triglycerides and glucose were measured fasting before and after intervention, as well as anti-psychotic efficacy scores such as PANSS score. P values were included. |
| **Was the precession of the estimate of the intervention or treatment effect reported?** | No confidence interval reported |
| **Do the benefits of the experimental intervention outweigh the harms and costs?** | Can’t tell. The combination of olanzapine and aripiprazole cost may be too high and alternatives approaches such as switching antipsychotic medication may be more suitable. |
| **Will the results help locally.** |  |
| **Can the results be applied to your local population?** | Can’t tell, don’t know if psychotic patients locally are similar to those in the study. Larger sample size required. |
| **Would the experimental intervention provide greater value to the people in your care than any of the existing interventions ?** | Can’t tell, as long-term, placebo-controlled trials are warranted to further assess the efficacy, safety, cost effectiveness, and metabolic benefit of the addition of aripiprazole to olanzapine-treated patients. There are practical considerations to implementing this treatment, such as cost and adverse reactions and clinical efficacy of treatment |

**Critical appraisal of study 6 using JBL case series checklist**

| Critical appraisal of study 6 |  |
| --- | --- |
| Were there clear criteria for inclusion in the case series? | No clear criteria for inclusion. Stud was a case series of 4 patients. All patients had a diagnosis of schizophrenia and were already on treatment of olanzapine. |
| Was the condition measured in a standard, reliable way for all participants included in the case series? | Yes, psychiatric symptoms were measured using the PANSS score for all patients and weight BMI were measured for all patients too. Fasting glucose, cholesterol both HDL and LDL and triglycerides were also measured. |
| Were valid methods used for identification of the condition for all participants included in the case series? | Yes, the DSM(IV) criteria was used in the diagnosis of all patients |
| Did the case series have consecutive inclusion of participants? | No |
| Did the case series have complete inclusion of participants? | Can’t tell |
| Was there clear reporting of the demographics of the participants in the study? | To some extent, age and sex are detailed, but ethnicity and geographic region are not. |
| Was there clear reporting of clinical information of the participants? | Yes, important clinical information such as BMI as reported for each patient. |
| Were the outcomes or follow up results of cases clearly reported? | Not all results were reported. Psychiatric symptoms were measured using the PANSS score for all patients and weight BMI were measured for all patients too. Fasting glucose, cholesterol both HDL and LDL and triglycerides were also measured but the exact figures were not provided |
| Was there clear reporting of the presenting site(s)/clinic(s) demographic information? | No |
| Was statistical analysis appropriate? | Yes, student t-tests were used as analysis. |

**Critical appraisal of study 7 using JBL case series checklist**

| Critical appraisal of study 7 |  |
| --- | --- |
| Were there clear criteria for inclusion in the case series? | Yes, Participants of this study were schizophrenic patients who were stable on olanzapine monotherapy on a daily dose of 10 mg for at least three months prior to the enrolment. The patients participated in this study were hoping to improve their BMI and glycaemic control. The exclusion criteria include any patients with dyslipidemia, diabetes or other endocrine disorder, patients with schizophrenia managed with polypharmacy, patients with renal and/or hepatic ailments, and pregnant or lactating mother. Schizophrenic patients managed with drugs known to affect glucose tolerance (β-adrenoceptor blockers, thiazides diuretics etc.) were also excluded. |
| Was the condition measured in a standard, reliable way for all participants included in the case series? | To some extent, measurements of fasting serum insulin (FSI), fasting blood glucose (FBG), C-peptide level and Hemoglobin A1C and BMI ere completed but a measurment of the symptoms of schiophrenia such as a PANSS score were not. |
| Were valid methods used for identification of the condition for all participants included in the case series? | Yes the DSM (V) was used. |
| Did the case series have consecutive inclusion of participants? | No |
| Did the case series have complete inclusion of participants? | No |
| Was there clear reporting of the demographics of the participants in the study? | To some extent, age and sex are detailed, but ethnicity and geographic region are not. |
| Was there clear reporting of clinical information of the participants? | No. It is detailed that all participants were schizophrenic patients who were stable on olanzapine monotherapy on a daily dose of 10 mg for at least three months prior to the enrolment, but there are no clinical details regarding their metabolic health sure as BMI. |
| Were the outcomes or follow up results of cases clearly reported? | Yes, measurements of fasting serum insulin (FSI), fasting blood glucose (FBG), C-peptide level and Hemoglobin A1C and BMI were taken. |
| Was there clear reporting of the presenting site(s)/clinic(s) demographic information? | No |
| Was statistical analysis appropriate? | Yes and completed. Continuous variables were represented as means and standard deviation (SD), while categorized variables in this study were represented as frequencies and percentages. Paired t t-test used for assessing the difference in glycemic control parameters before and after 8-weeks of using aripiprazole as an adjunctive therapy, whereas independent t t-test was used to analyze the gender difference in glycemic control parameters. Pvalues ≤ 0.05 were considered statistically significant throughout data analysis. Data were analyzed using Microsoft Excel 2013 and Graphpad prism version 8 |
